# Long-term survival in a patient with multiple metastatic gastric cancer treated with PTX plus emvolimab and disitamab vedotin: case report and treatment experience: A case report

**DOI:** 10.1097/MD.0000000000036927

**Published:** 2024-01-19

**Authors:** Yongjin Zhou, Meifeng Zhang, Li Dai, Zhiqiang Yan, Haibin Wang, Hongxin Yang, Xiangren Jin, Qian Wang

**Affiliations:** a Department of Gastrointestinal Surgery, Affiliated Hospital of Guizhou Medical University, Guiyang, Guizhou Province, China; b Guizhou Medical University, Guiyang, Guizhou Province, China; c Department of Outpatient Clinic, Affiliated Hospital of Guizhou Medical University, Guiyang, Guizhou Province, China.

**Keywords:** CTC (circulating tumor cell), envafolimab, locally advanced gastric cancer, neoadjuvant intraperitoneal and systemic chemotherapy, nutrition

## Abstract

**Rationale:**

Most Chinese patients with locally advanced gastric cancer at diagnosis have an overall 5-year survival rate of <50%. Surgical resection alone is not suitable for patients with locally advanced gastric cancer. Currently, comprehensive treatment is the focus of locally advanced gastric cancer.

**Patients concerns:**

The patient, a 56-year-old female, was admitted to the hospital because of “4 + months of double hydronephrosis found during a physical examination.” Who was admitted for computer tomography and gastroscopy examinations, and take pathological tissue specimens during endoscopic examination.

**Diagnoses:**

Computed tomography assessment indicated ulcerative gastric cancer with an abdominal implant, bladder, and bone metastases. An endoscopic examination revealed that the ulcer of the gastric angle was huge, and through relevant auxiliary examinations, the diagnosis of this disease is gastric cancer complicated with multiple metastases to bladder, rectum, lumbar spine, and peritoneum. Clinically diagnosed as cT4bN3M1.

**Interventions:**

The patient is currently undergoing first, second, and third line neoadjuvant therapy, combined with immunotherapy, targeted therapy, neoadjuvant intraperitoneal systemic chemotherapy, nutritional support, and other treatment plans.

**Outcomes:**

After 15 cycles of treatment, the progression-free survival had reached 15 months. The patient had an NRS2002 score of 1, an ECOG score of I, a quality of life score of 55, albumin of 35.27 g/L, and a decrease in abdominal and pelvic fluid accumulation and exudation compared to before.

**Lessons:**

We demonstrated high survival of almost 3 years in a patient with gastric cancer that was complicated by bone, peritoneal, rectal, and bladder metastases. The combination of immunotherapy, targeted therapy, and neoadjuvant intraperitoneal systemic chemotherapy, along with the maintenance of nutritional status and CTCs could be a valuable modality for the subsequent treatment and observation of similar patients.

## 1. Introduction

Gastric cancer is a common malignant tumor worldwide, with a relatively poor prognosis and a serious threat to human health. According to the statistics of the International Agency for Research on Cancer, there will be about 1 million new cases of gastric cancer in the world in 2020, and about 800,000 deaths due to gastric cancer, ranking fifth in the incidence rate of malignant tumors and third in the mortality rate.^[1]^ As the early symptoms of gastric cancer are not obvious and more than half of the affected Chinese population refuse gastroscopy, these patients are already in the progressive stage when they are diagnosed. The 5-year survival rate of patients with progressive gastric cancer is approximately 30% and the median survival period is less than a year.^[2]^ Among the numerous organs affected by metastases, the peritoneum is one of the most common sites and one of the most complex and difficult medical challenges faced by clinicians. Patients with this type of combined multimetastatic gastric cancer have poor survival, with a 5-year survival rate of <10%.^[3]^ The median survival is only 3.1 to 10.6 months, year survival is 16.0% to 40.7%^[4]^; owing to its anatomical location, it is difficult for drugs to cross the peritoneal-plasma barrier, and this barrier limits the attainment of the effective concentrations of chemotherapeutic drugs in the peritoneal cavity.^[5]^ Peritoneal metastases are found in 20% of patients with gastric cancer during the preoperative examination, and more than 50% of patients with stage T3 and T4 gastric cancer develop peritoneal metastases after radical gastric cancer surgery. The prognosis for these patients is extremely poor.^[6]^ Therefore, the treatment of patients with peritoneal metastasis of gastric cancer is essential yet a challenge for clinicians. At present, the main treatment method for patients with advanced gastric cancer and multiple metastases is still systemic chemotherapy, and the median overall survival (OS) of patients receiving conventional chemotherapy is about 12 months.^[7]^ Since the first targeted drug approved for gastric cancer, trastuzumab, was introduced, progress in the treatment of gastric cancer has stagnated for nearly a decade. In recent years, anti HER2 therapy and various biomarker targeted gastric cancer (GC) therapy have recently broken this trend.^[8]^ For example, anti-programmed cell death 1 antibodies have demonstrated impressive efficacy and prolonged survival in untreated microsatellite instability high (MSI-H)/defective mismatch repair (dMMR) mGC patients.^[9]^ Substantial breakthroughs in the treatment of gastric cancer have been achieved with novel anti-HER2 therapeutic agents, such as trastuzumab deruxtecan and disitamab vedotin.^[10]^ In our patient, we followed the neoadjuvant intraperitoneal systemic chemotherapy (NIPS) treatment plan advocated by Prof Zhu Zhenggang from Shanghai Ruijin Hospital. With the release of disitamab vedotin, it indicates that HER2 positive gastric cancer patients and HER2 low expression gastric cancer patients have considerable antitumor efficacy and tolerable safety. This has given hope to patients with advanced gastric cancer treatment. Blocking angiogenesis is a key strategy in GC therapy, including anti-vascular endothelial growth factor (VEGF) monoclonal antibodies, VEGF-binding proteins, and VEGF receptor tyrosine kinase inhibitors.^[11]^ Recent studies have confirmed that albumin-bound paclitaxel analogs can maintain high concentrations in the peritoneal cavity for a long time owing to their high molecular weights. In addition, after intravenous administration, the can effectively penetrate the peritoneal cavity and remain in the intraperitoneal cavity for up to 72 hours.^[12]^ Programmed cell death 1 (PD-1) is a t cell surface receptor that inhibits immune responses when bound to its ligand (PDL1 or PD-L2), which is present in normal human cells and cancer cells. PD-1 is an immunotherapeutic drug such as the target of Envafolimab. One of their applications is in the treatment of MSI-H tumors, in which microsatellite sequences accumulate mutations due to dMMR. Through the comprehensive application of these new drugs and innovative treatment methods, we have achieved surprising therapeutic results for a gastric cancer patient with advanced multiple metastases.

**Figure 1.**
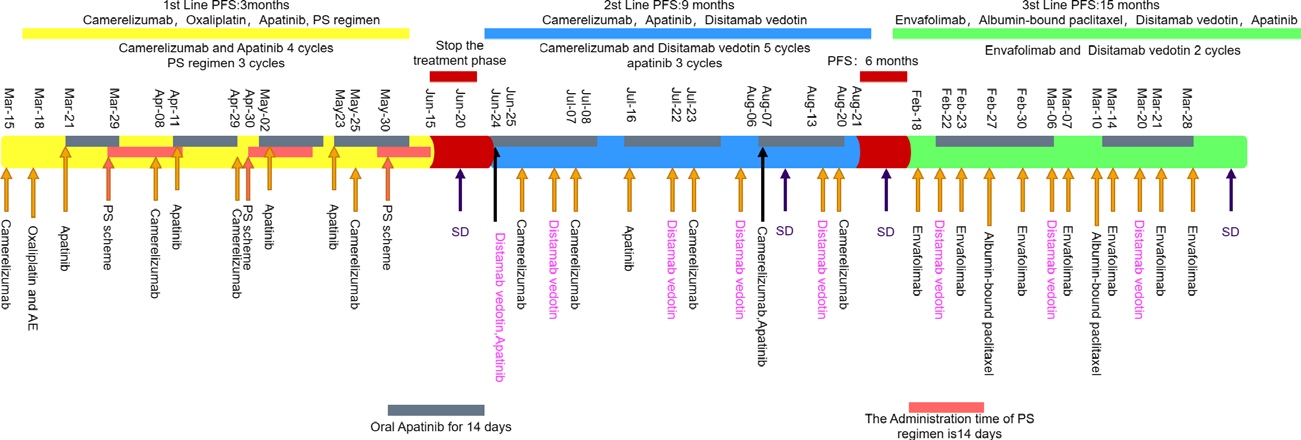
Treatment flow.

A 56-year-old female with gastric cancer complicated by multiple metastases of gastric cancer cT4bN3M1 (clinical stage IV) was diagnosed based on computed tomography (CT) on November 17, 2020, when she presented with bilateral hydronephrosis. First-line treatment (carrilizumab, SOX regimen, oral apatinib, and albumin-bound paclitaxel neoadjuvant intraperitoneal and systemic chemotherapy NIPS) was prescribed after discussion with a multidisciplinary team and expert consultation due to the presence of grade 3/4 Common Terminology Criteria for Adverse Events (CTCAE). The adverse reactions were not tolerated by the patient and treatment was suspended. This was followed up with second-line disitamab vedotin and the regimen was adjusted to disitamab vedotin intravenous drip, oral apatinib, and carrilizumab intravenous drip. The patient’s efficacy rating of this regimen until August 2021 was stable disease (SD).^[13]^ Our treatment strategy was found to be useful for a previous case. In February 2022, the patient’s regimen was changed to a third-line regimen and she was still alive with an SD efficacy rating and of nutritional status on the curative effect. Supplementing on-site nutrition when eating poorly. Figure 1 illustrate the entire treatment process. This study is aimed at serving as a reference for the subsequent treatment of patients with multiple metastases.

### 1.1. Consent

The patient signed an informed consent form for the publication of this case report and any accompanying images. The Ethics Committee of Guizhou Medical University Affiliated Hospital has abandoned ethical approval for this study as it is a case report of <3 patients (13 November, 2023).

## 2. Treatment process

*First-line treatment*: After 3 cycles of first-line carrilizumab, apatinib, and albumin-bound paclitaxel treatment. In detail: S-1 PO, 40 mg, for 14 days, Q3wks; PTX IVGTT 50 mg/m^2^ on days 1 and 8, intraperitoneal chemotherapy port PTX 20 mg/m^2^ on days 1 and 8, Q4wks. PTX was diluted in 1000 mL saline and ministered intraperitoneally via the intraperitoneal chemotherapy infusion port for 1 hour, and PTX was infused intravenously at the same time. Along with the antineoplastic therapy, nutritional support, traditional Chinese medicine, and other supporting treatments were.^[13]^

After the third cycle of PS treatment on June 6, 2021, there were CTCAE 3/4 grade adverse reactions. Among them, myelosuppression, gastrointestinal bleeding, and secondary bacterial and fungal infections are life-threatening. Symptoms of urinary incontinence, which is considered to be related to bladder metastasis, were also observed. The ECOG (Eastern Cooperative Oncology Group) score decreased to 4, and the quality of life score was 18. All antineoplastic therapies had to be suspended on June 15. The patient was in poor condition and could not bear the PS. After the first line of treatment, the tumor did not progress, which proved that our first line of treatment was effective. However, because of serious adverse reactions threatening the patient’s life, we had to stop the treatment. Since the patient was HER2 2+/FISH−, it was not advisable to administer Trastuzumab. So we switched to a treatment plan.

**Figure 2.**
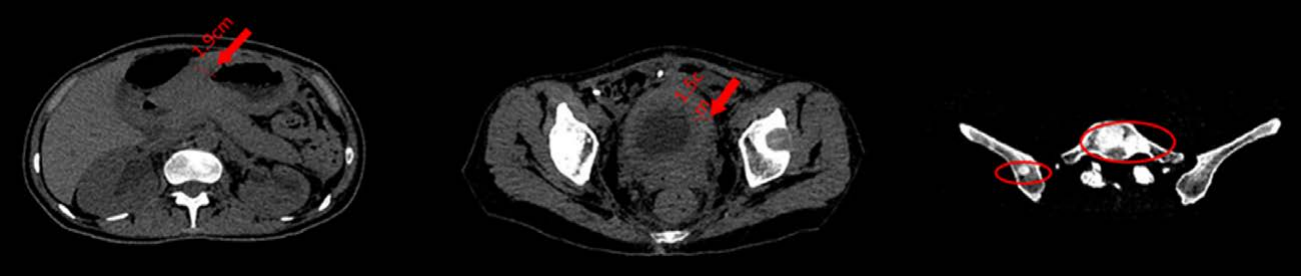
Changes in visible lesions on CT scans in November 2021. CT = computed tomography.

**Figure 3.**
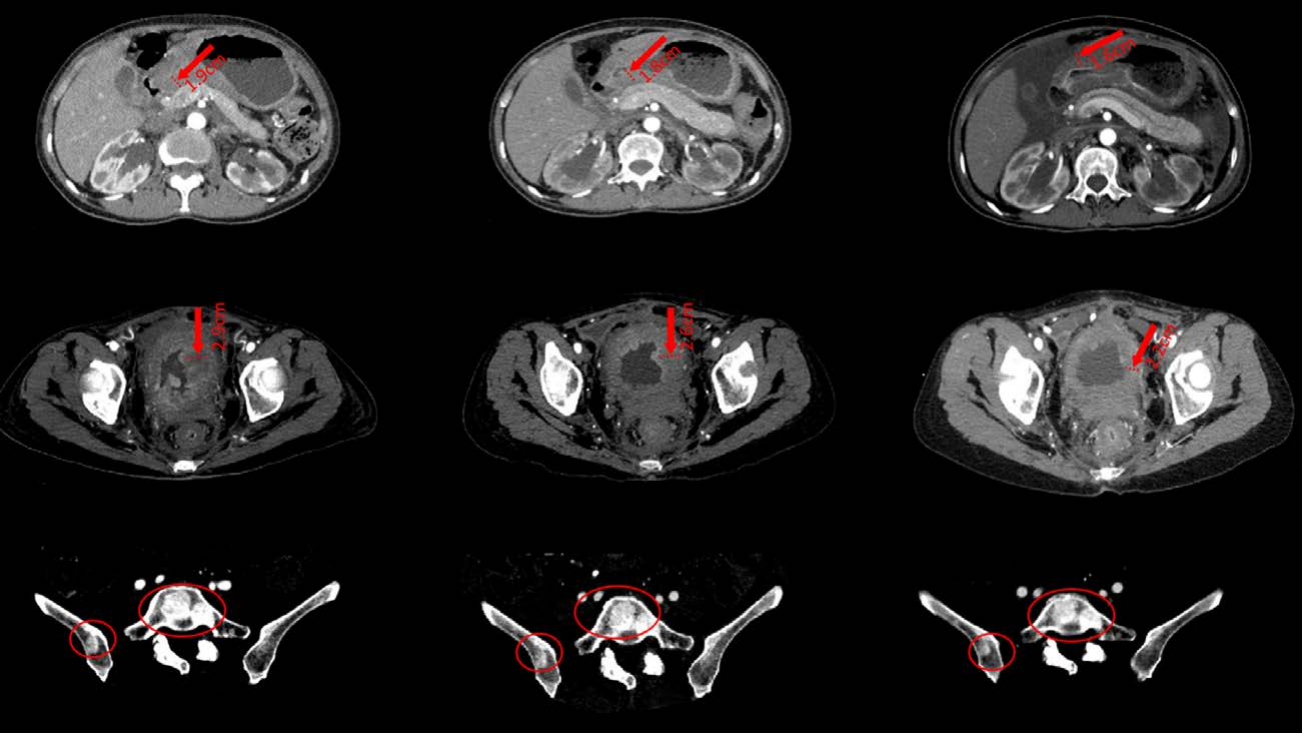
Changes in lesion seen on CT scan. CT = computed tomography.

*Second-line treatment*: Disitamab vedotin (IVGTT 100 mg Q2 wks), carrilizumab (IVGTT 200 mg Q3 wks), and oral apatinib (PO, 0.5 g, qd for 14 days, Q3 wks) was started on June 24, 2021, and continued until February 2022. CT scan on November 27, 2021 showed that pleural effusion was completely absorbed (Fig. 2); no progression of the gastric sinus wall or bladder wall thickening was noted (Fig. 3). The efficacy rating of the patient was still SD. During this period, we performed peripheral blood circulation tumor-cell testing (mesenchymal type 2, epithelial type 10, mixed type 4). During the treatment period, the NRS2002 score was 2 points, ECOG score was Level I, quality of life (QoL) score was 53 points, and the albumin level was between 35 and 40 g/L. At the same time, good nutritional status was maintained.

**Figure 4.**
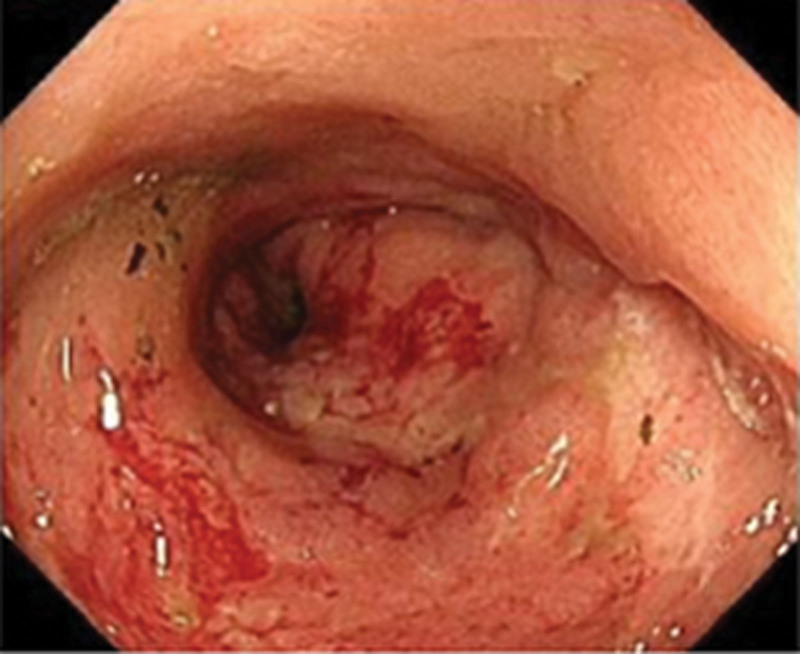
Changes in lesions during endoscopic examination. CT = computed tomography.

Only CTCAE grade 1/2 adverse reactions occurred during second-line treatment, confirming the feasibility of disitamab vedotin for HER2 2+ and 3+ advanced gastric cancer, regardless of the positive fluorescence in situ hybridization test. The second-line regimen continued for 9 months before the patient developed bloody stools in February 2022. A repeat endoscopy on February 17 showed an enlarged gastric giant ulcer with rectal metastases (Fig. 4), which forced us to adjust the treatment regimen. Circulating tumor cell (CTC) screening revealed 4 epithelial, 12 mesenchymal, and 6 mixed types. During the treatment period, the NRS2002 score was 2 points, ECOG score was Level I, and QoL score was 52 points; the nutritional score was also included. Albumin levels were between 35 and 40 g/L, and the nutritional status was good during the treatment period.

**Figure 5.**
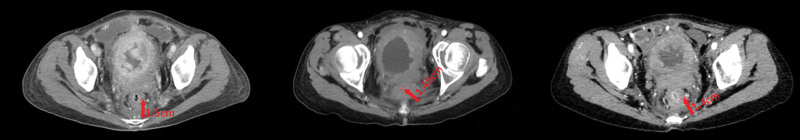
CT scan reveals changes in rectal metastases. CT = computed tomography.

**Figure 6.**
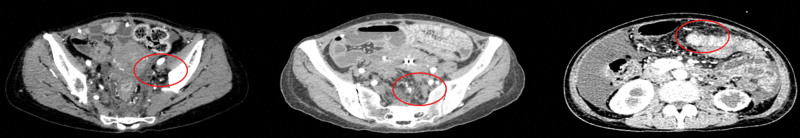
CT scan reveals changes in peritoneal metastasis lesions. CT = computed tomography.

*Third-line treatment*: It was decided that the regimen needed to be adjusted after a discussion with the multidisciplinary team, as this patient presented with an enlarged primary lesion and new peritoneal and rectal metastases (Figs. 5 and 6). Albumin-bound paclitaxel as first-line therapy was discontinued due to the inability to tolerate side effects. However, new peritoneal metastases appeared, and albumin-bound paclitaxel was continued to be infused intraperitoneally via a NIPS tube. Gastric and rectal lesions were retrieved for immunohistochemical analysis, which was suggestive of HER2 2+; thus, disitamab vedotin was continued. Moreover, the need to change PD-1 inhibitor–related drugs was considered; the patient’s programmed death-ligand 1 (PD-L1) test was negative, and the relevant literature indicates that patients who are PD-L1 negative can also benefit from PD-L1 inhibitor therapy.^[14,15]^

After discussions with the multidisciplinary team, a decision was made to use Envafolimab on a trial basis and to continue with apatinib. The specific regimen was changed and was as follows: Envafolimab (IH 200 mg Q1wks), disitamab vedotin (IVGTT 100 mg, Q2 wks), oral apatinib (PO, 0.5 g, qd for 14 days, Q3 wks), and albumin-bound paclitaxel (30 mg administered via the abdominal wall chemotherapy port). No serious adverse reactions occurred during treatment, and the NRS2002 score was 2, ECOG score was I, and QoL score was 52. Albumin was 35.27 g/L and prealbumin was 194 mg/L. Three cycles after changing the third-line regimen, the CT scan on May 9, 2022, showed that the patient had no significant progression of gastric, bladder, rectal, and abdominal metastases. She had an efficacy rating of SD, which confirmed that the third-line treatment was effective. CTC screening showed 4 epithelial, 3 mesenchymal, and 4 mixed types. At this time the NRS2002 score was 2, ECOG score was grade I, QoL score was 53, albumin level was 41 g/L, and prealbumin level was 162 mg/L. Patient’s poor eating during this period, extreme supplementation of albumin and enhanced parenteral nutrition was continued. The current conversion regimen was effective and the current regimen was continued. The patient had mild adverse effects after the third-line treatment and the efficacy was evaluated as SD. The current conversion regimen was maintained, and the patient developed hematuria twice during the period, which disappeared after the administration of hemostatic drugs.

On April 15, 2023, a CT review of gastric, bladder, rectal, and abdominal metastases showed no significant progression and an efficacy rating of SD. The NRS2002 score was 1, ECOG score was I, QoL score was 55, albumin level was 35.27 g/L, and prealbumin level was 162 mg/L, and the abdominopelvic fluid and exudate were reduced compared with the levels determined previously. Current CTC screening suggested 4 epithelial, 1 mesenchymal, and 4 mixed, and the CTCs were at relatively stable levels. At the time of submission of this case report, the patient was in good overall condition after 15 cycles of conversion therapy; she continues to receive third-line therapy.

## 3. Discussion

Currently, there is no internationally recognized standard treatment with significant efficacy for peritoneal metastasis of gastric cancer, and NIPS is still recommended in the National Comprehensive Cancer Network guidelines. NIPS combines the advantages of systemic chemotherapy and local abdominal treatment and has received a lot of attention in the treatment of peritoneal metastases from gastric cancer. In NIPS treatment, peritoneal chemotherapy differs from hyperthermic intraperitoneal chemotherapy (HIPEC) in which a specific thermostatic infusion containing chemotherapeutic agents is infused into the abdominal cavity for a certain period of time via circulating perfusion; in peritoneal chemotherapy, chemotherapeutic agents are injected into the pelvic floor of the abdominal cavity via a peritoneal chemotherapy pump left subcutaneously in the right iliac region of the patient and a catheter placed in the abdominal cavity. Contrary to HIPEC, NIPS is characterized by the ease of operation and administration, and is less painful and more acceptable to the patient. A peritoneal chemotherapy pump can be retained for 1 to 2 years, and multiple doses can be administered depending on the patient’s condition.^[16,17]^ In 2009 and 2012, Yonemura et al^[17,18]^ demonstrated the safety and efficacy of NIPS combined with cytoreductive surgery and concluded that NIPS conversion therapy is safe and effective in treating peritoneal metastases from gastric cancer. Additionally, NIPS combined with tumor cytoreductive surgery prolonged patient survival, especially in those with complete tumor regression, resulting in a significantly improved 5-year survival rate.^[19]^ NIPS combined with tumor cytoreduction surgery reduces the incidence of postoperative complications and mortality compared with HIPEC combined with tumor cytoreduction surgery. Kitayama et al improved the surgical protocol after NIPS conversion therapy and advocated radical gastrectomy.^[20,21]^ Recently, the results of a large number of clinical studies found that patients with gastric cancer combined with peritoneal metastases often do not have an indication for surgery and often have more appropriate surgical opportunities or even R0 resection after timely NIPS conversion therapy, prolonging survival time and improving the QoL; therefore, it shows potential as translational therapy in clinical applications.^[4]^

We formulated a personalized treatment plan based on changes in the patient’s condition, the latest global and relevant clinical studies, and after consultation with authoritative experts in the field of gastric cancer in China as well as our own clinical experience. First, in the third-line treatment, we will replace carolizumab in the second-line treatment with envafolimab. Although the patient was realistically negative for PD-L1 gene test and mismatch repair, we reviewed the relevant literature and found that patients who were PD-L1 negative could also benefit from using PD-1 inhibitors or PD-L1 inhibitors. An article evaluated the PD-L1 expression status of PD-L1 inhibitors in 53% of patients (n = 68). The 5-year survival rates of patients in the PD-L1 expression levels below 1%, 1% and above, and 50% and above subgroups were 20%, 23%, and 43%, respectively. The good efficacy and safety of Envafolimab were demonstrated in a study on 103 cancer cases. The objective effective rate was 42.7%, and the disease control rate was 66.0%. The duration of the 12 month remission rate was 92.2%. The median progression free survival period is 11.1 months. The OS rate at 12 months was 74.6%.^[22]^ These results indicate that even in patients with low or missing PD-L1 expression, there exists a certain population that can be considered for therapy with PD-1 inhibitors or PD-L1 inhibitors.^[14]^ We reviewed the relevant literature and reported a case study of a 76-year-old white male with a magnetic resonance imaging scan of the left hip showing a soft tissue lesion of the left iliac bone involving the acetabulum. Biopsy of the acetabular soft tissue mass showed hypofractionated adenocarcinoma, and positron emission tomography–CT showed partial pathological remission after 3 months of therapy with PD-1 inhibitors; progression was noted at review after 5 months of treatment, and the patient’s CT scan indicated complete pathological remission within 6 months during PD-L1 inhibitor replacement. After 35 cycles of PD-L1 inhibitor therapy, there were no immune-related adverse events, and sustained complete remission was noted.^[23]^ Although the mechanisms of action of PD-1 and PD-L1 inhibitors are similar, there are some differences in their efficacies and mechanisms of action. The efficacies of PD-1 and PD- L1 antibodies were compared using half-maximal effective concentration values obtained from functional assays. The half-maximal effective concentration of PD-L1 antibodies was significantly lower than that of PD-1 antibodies, suggesting that PD-L1 antibodies may be a more effective inhibitor than PD-1 antibodies.^[24]^ PD-L1 protein is an important transmodel immunoglobulin on the cell membrane that plays a crucial role in the formation of the tumor microenvironment and tumor immune escape. Blocking the PD1/PD-L1 signaling pathway can annul the inhibitory effect on lymphocytes. Although the PD-L1 gene test is realistically negative and the microsatellite is stable, we still add envafolimab, an innovative PD-L1 antibody drug developed in China, which combines a humanized single-domain PD-L1 antibody and a human immunoglobulin G1 Fc fragment covalently linked by an interchain disulfide bond.^[25]^ It enhances recognition of tumor cells by immune T cells in the body, which can bind PD-L1 protein and block its interaction with the PD-1 receptor, exert an inhibitory effect on the tumor via T cells through the PD-1/PD-L1 pathway, and enhance the antitumor activity of the immune system to kill the tumor. We reviewed the relevant literature and found that PD-1 inhibitors combined with chemotherapy were effective in treating patients with HER2-negative advanced gastric cancer, significantly increasing the OS rate of patients at 12 months by 57% compared with that achieved using chemotherapy alone,^[15]^ increasing the objective remission rate of up to 57.1%,^[26]^ and significantly prolonging the progression-free survival of patients by 36%.^[15]^ Drug safety and adverse effects that can be easily managed are some of the benefits for patients who are PD-L1 negative.^[14,15]^ Moreover, the structure of envafolimab differs from that of traditional antibodies such as pembrolizumab and nivolumab in that the antibody-binding region contains both heavy and light chains.^[27]^ Envafolimab is a heavy-chain antibody from camelids that has half the molecular weight of conventional antibodies. In addition, the antigen-binding region of envafolimab is the main bioactive region for its target-binding and blocking functions, competing with PD-1 mainly through a single surface loop consisting of 21 amino acids in the same plane on PD-L1. In conclusion, the unique structure of envafolimab provides a more flexible antigen-binding mechanism versus PD-1, with a stronger binding capacity to PD-L1 (~1000-fold), resulting in higher binding affinity and specificity.^[25]^ A phase II clinical trial of envafolimab (ClinicalTrials.gov: NCT03667170) was conducted at 25 clinical sites in China and enrolled 103 patients with MSI-H/ dMMR tumors. During treatment, the single drug envafolimab showed satisfactory therapeutic effects. Envafolimab exhibits good tolerance and safety in the treatment of advanced solid tumors.^[22]^ Irrespective of the cancer type, envafolimab was comparable with Pembrolizumab, Nivolumab, and Durvalumab in terms of efficacy and adherence. In terms of adverse events, the incidence of grade ≥ 3 treatment-emergent adverse events and immune-related adverse events associated with envafolimab was approximately 20% and 10%, respectively, and was roughly comparable with those of other PD-1/PD-L1 monoclonal antibodies.^[25]^ However, immune enterocolitis occurred in approximately 2% to 7% of patients treated with pembrolizumab, and immune pneumonia occurred in 1% to 5% of patients. Immune pneumonia or enterocolitis has not been observed in patients treated with envafolimab, and it is possibly related to the functional reserve between PD-1 and programmed death ligand 2.^[28,29]^ With excellent structural stability, low molecular weight, and strong tissue penetration, envafolimab antibodies represent a potential advancement in therapy as they can be easily administered subcutaneously; moreover, monodomain antibodies lacking immunoglobulin light chains are a possible alternative to full monoclonal antibodies. Although they tend to have a shorter half-life due to rapid renal clearance, they are more soluble and stable compared with all-monoclonal antibodies and, more importantly, they can penetrate tissues more rapidly. These properties are suitable for the subcutaneous administration of envafolimab antibodies.^[28]^ In addition, because it is administered subcutaneously, envafolimab monotherapy does not cause infusion reactions as seen with other immune checkpoint inhibitor treatments (0% vs 1% to 4%, respectively).Envafolimab may improve compliance and tolerability of immunotherapy in patients with advanced solid tumors.^[25]^ This is also an important reason why many patients are willing to use this drug. Good patient compliance also improves the QoL and survival to some extent. Angiogenesis is an important component of tumor growth, invasion, and metastasis,^[30,31]^ while vascular endothelial growth factor (VEGF) and its receptors, specifically VEGF receptor 2 (VEGFR2), are predominantly responsible for angiogenic signaling.^[32,33]^ Apatinib is a small molecule VEGFR inhibitor with China Food and Drug Administration (CFDA) approval for the treatment of advanced or metastatic chemotherapy-refractory GC. Apatinib improved median progression-free survival for 26 months and OS for 6.5 months versus placebo in Chinese patients with advanced gastric or gastroesophageal junction adenocarcinoma in the third line and beyond.^[34]^ Disitamab vedotin has been approved in China for the treatment of advanced or metastatic G/GEJ adenocarcinoma patients with overexpression of HER2, and has shown efficacy in our second and third line treatments. Our previous case report discussed the specific mechanism of action of dexamethasone.

Patients with malignancies have more serious nutritional problems caused by the disease itself and during cancer treatment. Malnutrition has negative effects on patients with cancer such as reducing the tolerance and efficacy of treatment,^[35]^ increasing the risk of clinical and surgical complications,^[36]^ prolonging hospital stays, and increasing medical costs. It is estimated that 10% to 20% of patients with cancer die because of malnutrition rather than cancer itself. Gastrointestinal malignancies are associated with a high risk of malnutrition.^[37]^ Active and effective nutritional therapy significantly improves clinical outcomes and complication rates of patients and shows benefits in prognosis and survival quality.^[38]^ Timely assessment of patients’ nutritional status and providing active nutritional support is important in improving treatment outcomes and reducing complications. High metabolism and stress in malignant tumors often lead to different degrees of malnutrition, and the nutritional risks of patients with cancer have been reported to be as high as 39% to 44%.^[39]^ The nutritional status of patients directly affects treatment outcomes and intraoperative and postoperative complication rates; moreover, it provides benefits for neoadjuvant chemotherapy. Thus, early nutritional intervention significantly improves treatment tolerability and patient survival.^[40]^ If the patient follows the prescribed diet, no enteral nutrition intervention is required. If the amount of ingested food is less than half of the normal amount of the required food, enteral nutrition should be provided orally. Nutritional status was assessed at the time of hospitalization. The literature indicates that the early implementation of nutritional-intervention programs for patients with cancer can significantly improve their nutritional status and prevent malnutrition.^[41,42]^ Accordingly, we paid special attention to the patient’s nutritional support during her treatment and she maintained an NRS2002 score of 1, ECOG score of I, and QoL score of 50 to 55, which was in the relatively satisfactory range during the treatment period. This may also be the reason why our patient exhibited good outcomes after chemotherapy and was able to continue with her treatment.

CTCs are an important bridge between the primary lesion and metastasis and play an important role in tumor metastasis. CTCs are shed from the primary lesion or metastasis and they metastasize through blood circulation. Most CTCs are attacked by the body’s immune cells and eliminated, whereas only those with high metastatic potential survive and develop distant metastasis. Some studies suggest that CTCs can be used to assess the prognosis and metastasis of patients with gastric cancer. We reviewed the patient’s CTCs several times during treatment and found that the number of interstitial CTCs was significantly higher in the presence of new lesions in our patient, who had positive CTCs because the primary tumor was not resected. Interstitial CTCs are related to the ability of tumor metastasis. The higher the number of interstitial CTCs, the higher the likelihood of tumor metastasis; therefore, CTCs can be regularly monitored in patients post-surgery and also in inoperable patients to determine tumor recurrence and progression.

In conclusion, we cannot deny the benefits of first- and second-line treatments. We demonstrated high survival of almost 3 years in a patient with gastric cancer that was complicated by bone, peritoneal, rectal, and bladder metastases. The combination of immunotherapy, targeted therapy, and NIPS, along with the maintenance of nutritional status and CTCs could be a valuable modality for the subsequent treatment and observation of similar patients.

## Acknowledgments

All authors have read the journal’s policy on conflicts of interest and authorship agreement.

## Author contributions

**Data curation:** Meifeng Zhang, Li Dai, Zhiqiang Yan, Hongxin Yang, Qian Wang, Xiangren Jin.

**Formal analysis:** Yongjin Zhou, Meifeng Zhang, Haibin Wang, Hongxin Yang, Qian Wang, Xiangren Jin.

**Investigation:** Yongjin Zhou, Meifeng Zhang, Li Dai, Qian Wang, Xiangren Jin.

**Methodology:** Yongjin Zhou, Meifeng Zhang, Li Dai, Zhiqiang Yan, Haibin Wang, Hongxin Yang, Qian Wang, Xiangren Jin.

**Supervision:** Xiangren Jin.

**Writing – original draft:** Yongjin Zhou, Qian Wang, Xiangren Jin.

**Writing – review & editing:** Yongjin Zhou, Hongxin Yang, Qian Wang, Xiangren Jin.
